# Negative cooperativity across β_1_-adrenoceptor homodimers provides insights into the nature of the secondary low-affinity CGP 12177 β1-adrenoceptor binding conformation

**DOI:** 10.1096/fj.14-265199

**Published:** 2015-04-02

**Authors:** Karolina Gherbi, Lauren T. May, Jillian G. Baker, Stephen J. Briddon, Stephen J. Hill

**Affiliations:** Cell Signalling Research Group, School of Life Sciences, University of Nottingham, Nottingham, United Kingdom

**Keywords:** dissociation, receptor dimerization, GPCR, allosterism

## Abstract

At the β_1_-adrenoceptor, CGP 12177 potently antagonizes agonist responses at the primary high-affinity catecholamine conformation while also exerting agonist effects of its own through a secondary low-affinity conformation. A recent mutagenesis study identified transmembrane region (TM)4 of the β_1_-adrenoceptor as key for this low-affinity conformation. Others suggested that TM4 has a role in β_1_-adrenoceptor oligomerization. Here, assessment of the dissociation rate of a fluorescent analog of CGP 12177 [bordifluoropyrromethane-tetramethylrhodamine-(±)CGP 12177 (BODIPY-TMR-CGP)] at the human β_1_-adrenoceptor expressed in Chinese hamster ovary cells revealed negative cooperative interactions between 2 distinct β_1_-adrenoceptor conformations. The dissociation rate of 3 nM BODIPY-TMR-CGP was 0.09 ± 0.01 min^−1^ in the absence of competitor ligands, and this was enhanced 2.2- and 2.1-fold in the presence of 1 µM CGP 12177 and 1 µM propranolol, respectively. These effects on the BODIPY-TMR-CGP dissociation rate were markedly enhanced in β_1_-adrenoceptor homodimers constrained by bimolecular fluorescence complementation (9.8- and 9.9-fold for 1 µM CGP 12177 and 1 µM propranolol, respectively) and abolished in β_1_-adrenoceptors containing TM4 mutations vital for the second conformation pharmacology. This study suggests that negative cooperativity across a β_1_-adrenoceptor homodimer may be responsible for generating the low-affinity pharmacology of the secondary β_1_-adrenoceptor conformation.—Gherbi, K., May, L. T., Baker, J. G., Briddon, S. J., Hill, S. J. Negative cooperativity across β_1_-adrenoceptor homodimers provides insights into the nature of the secondary low-affinity CGP 12177 β1-adrenoceptor binding conformation.

The β_1_-adrenoceptor has been reported to exist in 2 active conformations (1–6), although the nature of the secondary β_1_-adrenoceptor conformation has been the subject of speculation for many years (7–11). The endogenous ligands adrenaline and noradrenaline exhibit their agonist effects through the orthosteric catecholamine site of the β_1_-adrenoceptor. These agonist responses are antagonized by β-blockers such as CGP 20712A, CGP 12177, and propranolol with high affinity (6, 12–14). However, CGP 12177 (15) was also found to exert partial agonist effects at 100-fold higher concentrations than were needed to antagonize catecholamine-mediated agonist responses (4, 12).

According to classic receptor theory, the EC_50_ of a partial agonist is expected to be similar to its binding affinity. The discrepancy between these 2 values for CGP 12177 and also other β-adrenoceptor ligands, such as pindolol, led to the classification of nonconventional β-adrenoceptor agonists (16). Furthermore, the CGP 12177 agonist effect appeared resistant to β-blocker antagonism at concentrations normally sufficient to block catecholamine-mediated agonist effects (4, 6, 12, 14, 17). As such, the affinities of a range of β-adrenoceptor antagonists have been reported to be ≥1 order of magnitude lower when inhibiting responses mediated by CGP 12177 at the β_1_-adrenoceptor compared with those mediated by catecholamines in both animal and human tissue preparations (10, 14, 18). It is noteworthy that the use of recombinant cell systems and cardiac tissue isolated from β_2_- and β_1_-/β_2_-adrenoceptor knockout mice clearly showed that the β_1_-adrenoceptor alone was responsible for the observed CGP 12177 pharmacology (1, 3, 4). This led to the proposal that there are 2 active conformations of the β_1_-adrenoceptor: a primary high-affinity endogenous catecholamine site and a secondary low-affinity CGP 12177 site (1, 4).

Site-directed mutagenesis has been used in previous studies to investigate the nature of the secondary low-affinity β_1_-adrenoceptor conformation (7, 11). Initial work suggested some overlap of the 2 proposed β_1_-adrenoceptor binding conformations (7). However, more recently, Baker *et al.* (11) demonstrated that residues L195 and W199 in transmembrane domain (TM)4 are essential for the secondary β_1_-adrenoceptor conformation (11). Furthermore, TM4 may have a role in oligomerization (19), because the formation of β_1_-adrenoceptor homodimers has been reported previously (20–22), and an important interface for this appears to be between TM4 and TM5 (19).

A β_1_-adrenoceptor homodimer complex would possess 2 structurally identical orthosteric β_1_-adrenoceptor sites, to which ligands would be expected to bind with similar affinities. However, negative cooperative interactions between the 2 orthosteric β_1_-adrenoceptor binding sites may provide an explanation of the lower affinity observed for the secondary β_1_-adrenoceptor protomer, if indeed this occurs as a dimer (23). Negative cooperativity across a homodimer interface has previously been described for the human A_3_ adenosine receptor (23). In this example, negative cooperativity was demonstrated in single living cells by following the impact of orthosteric unlabeled ligands binding to one protomer of an A_3_-homodimer on the dissociation of a fluorescently labeled agonist (which was enhanced) from the orthosteric site of the other A_3_-receptor protomer (23).

We previously showed that the fluorescent CGP 12177 analog bordifluoropyrromethane-tetramethylrhodamine-(±)CGP 12177 (BODIPY-TMR-CGP) can be used to label both conformations of the β_1_-adrenoceptor (24). In this study, we used this fluorescent CGP 12177 analog to investigate the potential for allosteric interactions across a homodimer interface of the β_1_-adrenoceptor using kinetic measurements of BODIPY-TMR-CGP binding in single living cells.

## MATERIALS AND METHODS

### Materials

Cell culture plastics were purchased from Thermo Fisher Scientific (Loughborough, United Kingdom), and cell culture reagents were from Sigma-Aldrich (Gillingham, United Kingdom) except for fetal calf serum, which was obtained from PAA Laboratories (Pasching, Austria). Lipofectamine 2000 transfection reagent and Opti-MEM medium were from Invitrogen (Paisley, United Kingdom), and SNAP-Surface 488 was from New England Biolabs (Ipswich, MA, USA). BODIPY-TMR-CGP was from Molecular Probes (Leiden, The Netherlands), and unlabeled CGP 12177 and propranolol were from Tocris Cookson (Avonmouth, Bristol, United Kingdom). All other reagents were from Sigma Chemicals (Poole, United Kingdom).

### Cell culture

Chinese hamster ovary (CHO)-K1 cells were used for all transient transfections. CHO-K1 cells stably expressing the secreted placental alkaline phosphatase reporter gene under the transcriptional control of a 6-cAMP response element promoter (CHO-CS cells) were used as a control, as appropriate. CHO-CS cell lines either expressing human wild-type β_1_-adrenoceptors (CHO-β_1_ cells; 1147 fmol/mg protein) (6) or human β_1_-adrenoceptors containing 11 amino acid mutations (G177V, L178I, V179I, C180L, T181M, A184I, I185V, A187G, V189T, L195Q, and W199Y that convert TM4 to the equivalent residues in the β_2_-adrenoceptor; CHO-β_1_TM4 cells) (11) were used. CHO-K1, CHO-CS, CHO-β_1_, and CHO-β_1_TM4 cells were grown at 37°C in CHO growth medium [DMEM/Ham's nutrient mixture F12 containing 10% (v/v) fetal calf serum and 2 mM l-glutamine] in a humidified 5% CO_2_/95% air atmosphere.

### Generation of β_1_-adrenoceptor constructs

The β_1_-yellow fluorescent protein (YFP)_N_ and β_1_-YFP_C_ receptor constructs were generated by fusing either the N-terminal fragment of YFP (YFP_N;_ amino acids 1–155) or the C-terminal fragment of YFP (YFP_C_; amino acids 156–239) to the C-terminal end of the full-length wild-type human β_1_-adrenoceptor. The SNAP-β_1_ construct was generated by fusing the SNAP-tag (New England Biolabs, Ipswich, MA, USA) to the N-terminal end of the wild-type human β_1_-adrenoceptor. The D138A mutation (7) was introduced into the β_1_-YFP_C_ and the SNAP-β_1_ sequence using the QuikChange site-directed mutagenesis kit (Agilent Technologies, Cheshire, United Kingdom). All sequences were confirmed by DNA sequencing. All receptor constructs were subcloned into pcDNA3.1 vectors.

### SNAP-tag labeling and confocal imaging

Confocal microscopy was performed using a Zeiss LSM710 laser scanning microscope with a ×40 1.3 NA oil immersion lens. CHO-K1 cells were grown to 70% confluence in 8-well Labtek borosilicate chambered-cover glasses (Nalgene Nunc International, Fisher Scientific) and transiently transfected with SNAP-β_1_ or SNAP-β_1D138A_ recombinant DNA using Lipofectamine 2000 and Opti-MEM medium according to the manufacturer’s instructions. The next day, a 1 µM concentration of the benzyl-guanine labeled SNAP-tag substrate SNAP-Surface 488 (BG-488) was prepared in fresh cell culture medium, added to these cells, and incubated in the dark for 30 min (room temperature). The cells then were washed twice in imaging buffer (147 mM NaCl, 24 mM KCl, 1.3 mM CaCl_2_, 1 mM MgSO_4_, 10 mM 4-(2-hydroxyethyl)-1-piperazineethanesulfonic acid, 2 mM sodium pyruvate, 1.43 mM NaHCO_3_ 4.5 mM d-glucose, pH 7.4). After this, 3 nM BODIPY-TMR-CGP was added to the cells in the dark for 10 min (room temperature), after which the cells were imaged immediately (1024 × 1024 pixels, averaging at 4 frames, pinhole diameter 1 airy unit); 561 nm diode and 488 nm argon lasers were used to excite BODIPY-TMR-CGP and BG-488, respectively. A variable spectral detection system was used to capture emission at 565–605 and 495–535 nm, respectively. Confocal settings for laser power, offset, and gain were kept constant throughout each experiment set.

### BODIPY-TMR-CGP kinetic studies using the confocal perfusion system

#### BODIPY-TMR-CGP association and dissociation kinetics

Live cell fluorescence imaging was performed on the Zeiss LSM510 laser scanning confocal microscope using a Zeiss Plan-Neofluar ×40 1.3 NA oil-immersion objective in conjunction with a temperature-controlled (37°C) perfusion system to allow the visualization and quantification of BODIPY-TMR-CGP dissociation kinetics under infinite dilution (ID) conditions in single living cells (25). Kinetic experiments were performed as described by May *et al.* (25). In brief, cells were grown to near confluence on 32 mm glass coverslips in 6-well plates 1 d prior to experimentation. On the day of experimentation, the coverslip was placed into a tightly closed imaging chamber on a heated (37°C) microscope stage, where it was connected to tubes to facilitate the flow of imaging buffer through the imaging chamber in the absence and presence of BODIPY-TMR-CGP and/or unlabeled ligands at a constant flow rate of ≥4 ml/min.

For association and dissociation kinetic experiments of 10–100 nM BODIPY-TMR-CGP in CHO-β_1_ and CHO-CS cells, the cells were exposed to imaging buffer only (30 s baseline fluorescence recording), then BODIPY-TMR-CGP (4.5 min association), and followed again by imaging buffer only (4.5 min dissociation). BODIPY-TMR-CGP was excited using a 543 nm helium-neon laser with emission collected through a 565 nm long-pass filter every 3 s throughout each experiment (512 × 512 pixels, averaging at 2 frames). The pinhole diameter (1 airy unit), laser power (2%), offset, and gain remained constant between the 3 BODIPY-TMR-CGP concentrations and the 2 cell lines used. Membrane-associated BODIPY-TMR-CGP fluorescence was measured by drawing regions of interest (ROIs) around the membranes of 10 single cells of each imaged coverslip, and changes in the average pixel intensity values for each ROI over time were analyzed to obtain association and dissociation rates for each experiment.

#### Measuring the influence of unlabeled ligands on the BODIPY-TMR-CGP dissociation rate

Live cell fluorescence imaging using 3 nM BODIPY-TMR-CGP was performed on the Zeiss LSM710 laser scanning confocal microscope using a Zeiss Plan-Neofluar ×40 1.3 NA oil-immersion objective in conjunction with a perfusion system as described above for the Zeiss LSM510 laser scanning confocal microscope. For association and dissociation kinetic experiments using 3 nM BODIPY-TMR-CGP in CHO-β_1_ and CHO-CS cells, the cells were exposed to imaging buffer only (30 s baseline fluorescence recording), then BODIPY-TMR-CGP (4 min association), and followed again by imaging buffer only (4 min dissociation). Influences of unlabeled ligands on the BODIPY-TMR-CGP dissociation rate in CHO-β_1_ cells were determined by perfusion of imaging buffer (30 s baseline read), 3 nM BODIPY-TMR-CGP (4 min association), and imaging buffer (4 min dissociation) in the absence or presence of CGP 12177 (0.01–10 µM) or propranolol (0.1–10 µM).

In experiments using CHO-β_1_TM4 cells, the cells were first exposed to 3 nM BODIPY-TMR-CGP for 3.5 min in a 6-well plate prior to placing the coverslip into the imaging chamber to achieve a significant but low level of labeling of the receptor. Once placed onto the microscope stage, the cells were perfused with BODIPY-TMR-CGP (30 s baseline), before perfusing imaging buffer in the absence or presence of 1 µM CGP 12177 or 1 µM propranolol (dissociation).

In bimolecular fluorescence complementation (BiFC) experiments, CHO-K1 cells were seeded onto coverslips on day 1 and transiently transfected with YFP_N_- and YFP_C_-tagged β_1_-adrenoceptor recombinant DNA (750 ng total) using Lipofectamine 2000 and Opti-MEM medium according to the manufacturer’s instructions on day 2. The following day, the transfection medium was removed and replaced with fresh CHO growth medium, before the cells were placed back into the cell culture incubator (37°C, 5% CO_2_/95% air atmosphere). After ∼6 h, the cells were moved into a 30°C incubator overnight (5% CO_2_/95% air atmosphere) to allow the maturation of the fluorophore following correct protein folding (26). On day 4, the cells were used for experimentation, and the dissociation of 3 nM BODIPY-TMR-CGP in the absence and presence of unlabeled ligands was determined as described above.

BODIPY-TMR-CGP and YFP (when used) were excited using a 561 nm diode and 488 argon laser, respectively, every 2 s throughout each experiment (512 × 512 pixels, averaging at 2 frames). A variable spectral detection system was used to capture BODIPY-TMR-CGP and YFP emission at 565–605 and 495–535 nm, respectively. The pinhole diameter (1 airy unit) and laser power (5%) remained constant between all experiments, but the gain and offset were adjusted for each experiment for optimal detection of 3 nM BODIPY-TMR-CGP, and kinetic data were expressed in percentage fluorescent intensity to allow data to be grouped and compared. ROIs were drawn around the membranes of 3–10 single cells of each imaged coverslip, and changes in average pixel intensity values for each ROI over time were analyzed to obtain BODIPY-TMR-CGP dissociation rates for each experiment. In BiFC experiments, membrane-associated BODIPY-TMR-CGP fluorescence was measured by drawing ROIs around membranes of cells that were identified to express BiFC-constrained homodimers by examination of the YFP fluorescence.

### Data analysis

GraphPad Prism 5.0 (GraphPad Software, San Diego, CA, USA) was used to fit all data presented in this study. Association kinetic data were fitted using the following monoexponential association equation:
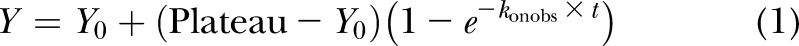
where *Y_0_* is the level of BODIPY-TMR-CGP binding at time *t* = 0 (*i.e.,* baseline fluorescence), Plateau is the level of BODIPY-TMR-CGP binding at infinite time, and *k*_onobs_ is the rate of observed association.

BODIPY-TMR-CGP fluorescence intensities measured at time *t*_4.5min_ in CHO-β_1_ cells and CHO-CS cells were plotted against the BODIPY-TMR-CGP concentrations used and fitted to a 1-site total binding saturation equation (Eq. 2) or a nonspecific binding linear regression equation (Eq. 3), respectively
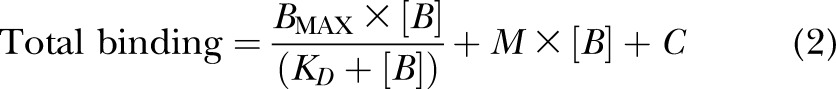

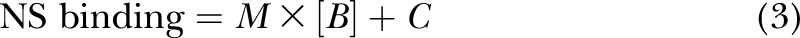
where *B*_MAX_ is the maximum specific BODIPY-TMR-CGP binding, [*B*] is the BODIPY-TMR-CGP concentration, *K_D_* is the BODIPY-TMR-CGP concentration that achieves 50% specific binding, *M* denotes the slope of the linear regression component, and *C* is the background fluorescence intensity.

The dissociation kinetic data were analyzed using the following monoexponential decay equation, if nonspecific binding of <10% of total binding was observed for a given BODIPY-TMR-CGP concentration
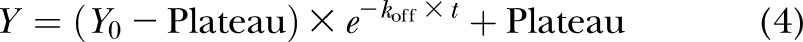
where *Y*_0_, Plateau, and *t* are the same as defined above with *t*_0_ representing the start of dissociation in *Y*_0_ (*i.e.,* the binding of BODIPY-TMR-CGP at time zero). The *k*_off_ is the dissociation rate of BODIPY-TMR-CGP. Where a level of nonspecific binding >10% of total binding was observed, the dissociation kinetic data were fitted to a 2-phase exponential decay function
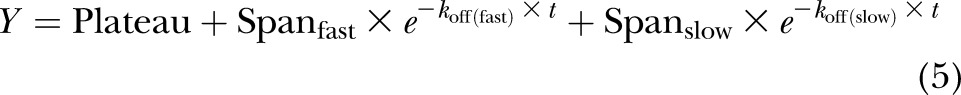
where Plateau and *t* are as defined above, and Span_fast_ and Span_slow_ represent the proportion of *Y*_0_-Plateau accounted for by the fast (*k*_off(fast)_) and slow (*k*_off(slow)_) dissociation rate, respectively. Within this analysis, *k*_off(fast)_ and Plateau was constrained to the average rate of dissociation and the average Plateau (in %) reached by BODIPY-TMR-CGP in control CHO-CS cells (*i.e.,* cell not expressing the receptor of interest to determine the nonspecific BODIPY-TMR-CGP binding component). Equation 6 was then used to calculate the association rate constants (*k*_on_) using the *k*_onobs_ and *k*_off(slow)_ determined above for each BODIPY-TMR-CGP concentration [*B*]
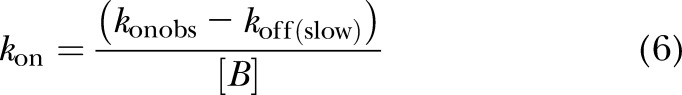
The negative logarithms of the equilibrium dissociation constant (*pK_D_*) were obtained using the above determined kinetic parameters in the following equation:
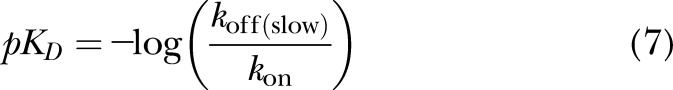
Experiments investigating the kinetic parameters of 3 nM BODIPY-TMR-CGP binding to CHO-β_1_ cells were analyzed using a global fit of its association (Eq. 8a) and dissociation traces (Eq. 8b)
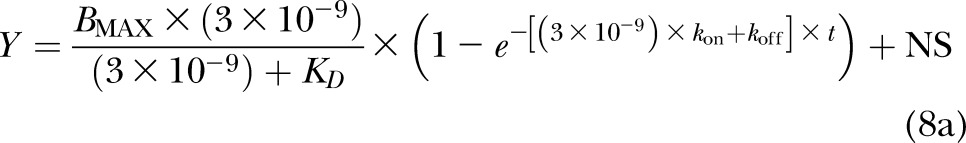

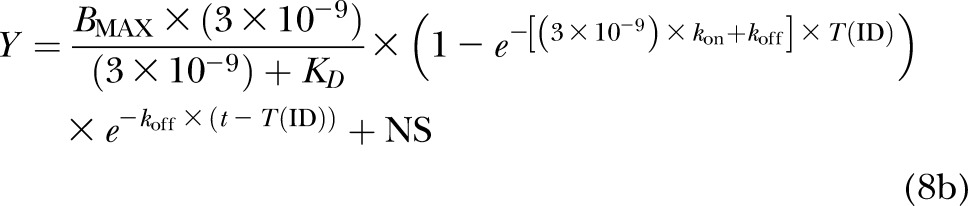
where *B*_MAX_ is the maximum specific BODIPY-TMR-CGP binding, *K_D_* is the BODIPY-TMR-CGP concentration that achieves 50% specific binding, NS is nonspecific binding, *T*(ID) is the time at which infinite dilution was commenced, and *k*_on_, *k*_off,_ and *t* are as described above.

Concentration-dependent cooperative effects of unlabeled ligands CGP 12177 and propranolol on the dissociation rate of 3 nM BODIPY-TMR-CGP were fit to the following equation:
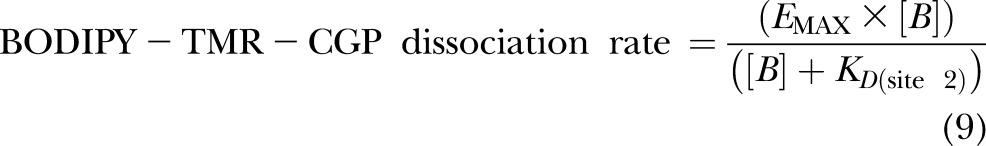
where *E*_MAX_ is the maximal increase in BODIPY-TMR-CGP dissociation rate, [*B*] is the concentration of unlabeled ligand, and *K_D_*_(__site__2)_ is the concentration of the unlabeled ligand that achieves 50% binding to a secondary binding site that exerts cooperative effects on the BODIPY-TMR-CGP dissociation from the primary binding site. A measure of cooperativity between 2 binding sites is provided by the cooperativity factor α, which was calculated using the following equation:
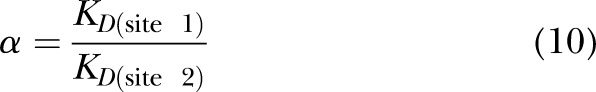
where *K_D_*_(__site__1)_ is the equilibrium dissociation constant of a given ligand for the primary (site 1) binding site of an unbound receptor, and *K_D_*_(__site__2)_ is the equilibrium dissociation constant of the same ligand for the secondary (site 2) binding site of a ligand-bound (at site 1) receptor.

All data are represented as means ± sem from *n* separate experiments. Statistical analysis was performed where appropriate and as detailed in the text, with *P* < 0.05 reflecting statistical significance.

## RESULTS

### Kinetic parameters of BODIPY-TMR-CGP at the human β_1_-adrenoceptor

**Figure 1.**
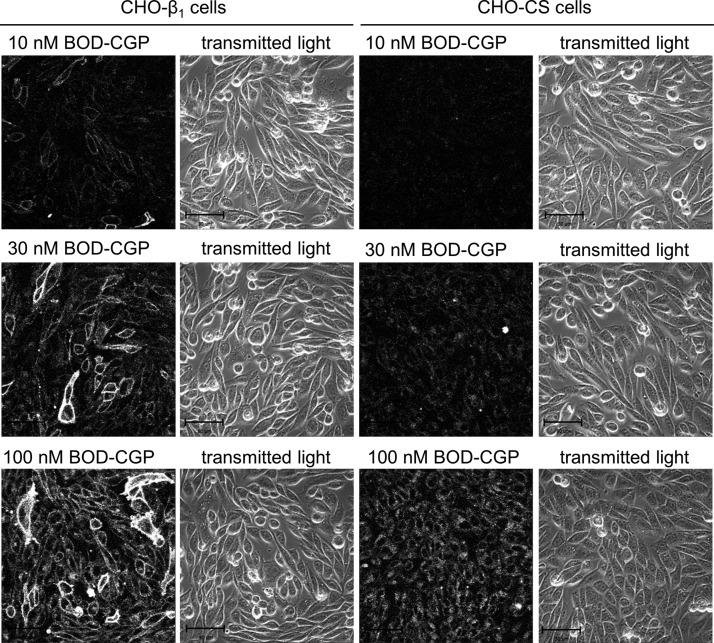
Concentration-dependent increase of BODIPY-TMR-CGP binding levels in CHO-β_1_ and CHO-CS cells. Confocal images of 10, 30, and 100 nM BODIPY-TMR-CGP (BOD-CGP) binding levels following 4.5 min association measured in CHO-β_1_ and CHO-CS cells. The microscope settings were kept constant for the recordings of fluorescence intensities of all 3 BODIPY-TMR-CGP concentrations in both CHO-β_1_ and CHO-CS cells to allow for direct comparison of the BODIPY-TMR-CGP binding levels. Images are representatives of a total of 5, 6, and 4 separate experiments (for 10, 30, and 100 nM BODIPY-TMR-CGP, respectively) using CHO-β_1_ cells, and 3, 6, and 3 separate experiments (for 10, 30, and 100 nM BODIPY-TMR-CGP, respectively) using CHO-CS cells. Scale bars, 50 μm.

**Figure 2.**
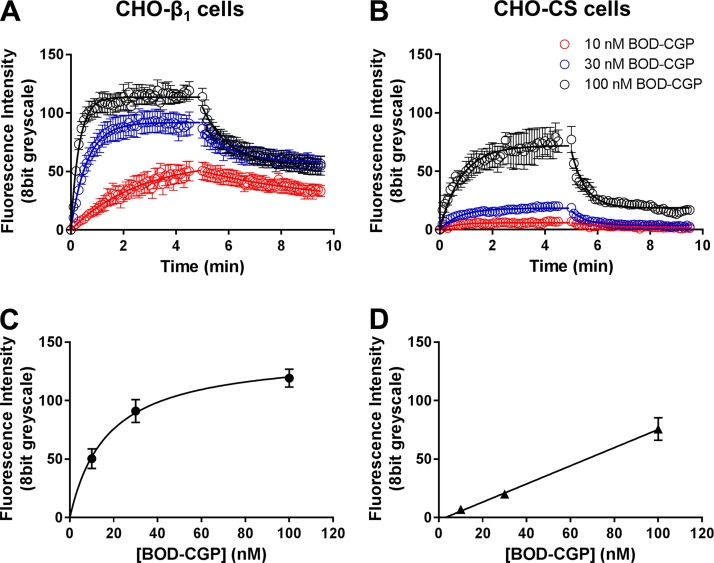
Characterization of BODIPY-TMR-CGP binding properties in CHO-β_1_ and CHO-CS cells. The fluorescence intensity of 10, 30, and 100 nM BODIPY-TMR-CGP (BOD-CGP) was monitored every 3 s (shown for every 6 s for better visualization) in (*A*) CHO-β_1_ and (*B*) CHO-CS cells to determine association rates during BODIPY-TMR-CGP perfusion (first 4.5 min), and dissociation rates during perfusion of imaging buffer only (subsequent 4.5 min). The fluorescence intensity values measured in (*C*) CHO-β_1_ and (*D*) CHO-CS cells following 4.5 min association of 10, 30, and 100 nM BODIPY-TMR-CGP were plotted against the corresponding BODIPY-TMR-CGP concentration and highlight the saturable binding of BODIPY-TMR-CGP to CHO-β_1_ but not CHO-CS cells at the concentrations used. Data shown are means ± sem of 3–6 separate experiments per BODIPY-TMR-CGP concentration in each cell line. Each experimental replicate reflects the average fluorescent intensity of the plasma membrane of 10 cells. Summary data and statistical analysis are provided in Table 1.

The association and dissociation of 10, 30, and 100 nM BODIPY-TMR-CGP at the human β_1_-adrenoceptor were examined in CHO-β_1_ and CHO-CS cells to determine total and nonspecific binding levels, respectively. Using the same microscope settings for all BODIPY-TMR-CGP concentrations in both cell lines allowed direct comparison of fluorescence intensities and thus BODIPY-TMR-CGP binding levels (**Fig. 1**). The fluorescence intensity levels increased with increasing BODIPY-TMR-CGP concentrations in both CHO-β_1_ and CHO-CS cells; however, the nonspecific cell membrane-associated fluorescence intensity in CHO-CS cells was markedly lower than the total binding cell membrane-associated fluorescence intensity measured in CHO-β_1_ cells (**Fig. 2*A, B***). A plot of the binding levels against BODIPY-TMR-CGP concentration was best described by a saturable and linear component following 4.5 min association in CHO-β_1_ cells (Fig. 2*C*). In contrast, the increase in BODIPY-TMR-CGP binding levels with increasing concentration of fluorescent label in CHO-CS cells was best described by a linear relationship (Fig. 2*D*), which is characteristic of nonspecific binding components. Interestingly the nonspecific binding component appeared smaller in the cells expressing the human β_1_-adrenoceptor (Fig. 2*C*).

The association and dissociation traces obtained in CHO-CS could only be accurately analyzed for 30 and 100 nM BODIPY-TMR-CGP, and revealed rapid observed association (*k*_onobs_) and dissociation (*k*_off_) rates (**Table 1**), which provides further evidence that BODIPY-TMR-CGP binding interactions in CHO-CS were nonspecific (27).

**TABLE 1.** Kinetic parameters of 10, 30, and 100 nM BODIPY-TMR-CGP at CHO-β_1_ and CHO-CS cells

BODIPY-TMR-CGP	*k*_onobs_ (min^−1^)	*k*_off(fast)_ (min^−1^)	*k*_off(slow)_ (min^−1^)	*k*_on_ (×10^7^ M^−1^⋅min^−1^)	*pK_D_*	*n*
CHO-CS (nM)						
10	NA	NA				3
30	1.40 ± 0.26	2.46 ± 0.48				6
100	1.62 ± 0.13	2.47 ± 0.13				3
CHO-β_1_ (nM)						
10	0.63 ± 0.08	NA	0.10 ± 0.01	5.29 ± 0.79	8.71 ± 0.05	5
30	1.64 ± 0.08*	2.47	0.09 ± 0.01	5.17 ± 0.28	8.77 ± 0.08	6
100	3.08 ± 0.11^*^^,^^#^	2.47	0.14 ± 0.02^*^^,^^#^	2.94 ± 0.12^*^^,^^#^	8.32 ± 0.06^*^^,^^#^	4

Data are means ± sem with *n* representing the number of separate experiments carried out. In each experiment, ROIs were drawn around the membrane of 10 cells. NA, not applicable. *Statistical significance (*P* < 0.05) from the value determined for 10 nM and ^#^*P* < 0.05 from 30 nM BODIPY-TMR-CGP in CHO-β_1_ cells (1-way ANOVA followed by Tukey’s multiple comparisons test).

**Figure 3.**
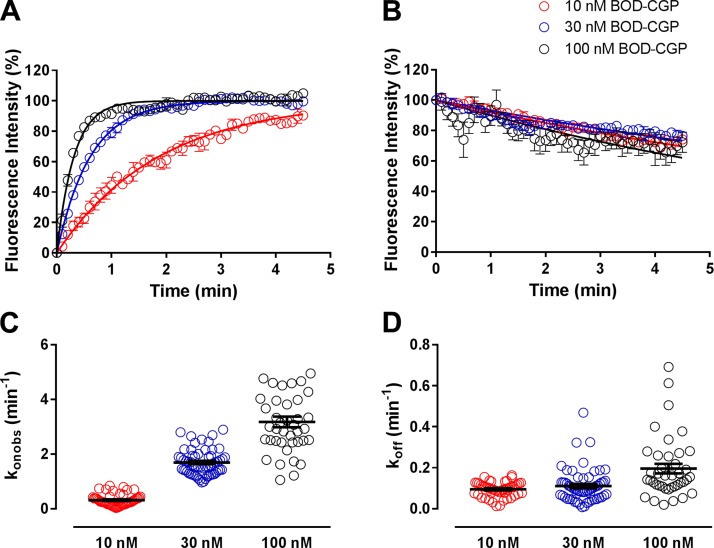
Association and dissociation kinetics of 10, 30, and 100 nM BODIPY-TMR-CGP in CHO-β_1_ cells. The fluorescence intensity of 10, 30, and 100 nM BODIPY-TMR-CGP (BOD-CGP) during perfusion of BODIPY-TMR-CGP (association) and imaging buffer only (dissociation) was monitored every 3 s (shown for every 6 s for better visualization). *A* and *B*) Normalized association (*A*) and dissociation data (*B*) of BODIPY-TMR-CGP (BOD-CGP) in CHO-β_1_ cells are means ± sem of 5, 6, and 4 separate experiments for 10, 30, and 100 nM BODIPY-TMR-CGP, respectively. Association data were normalized to the predicted maximum binding level of each BODIPY-TMR-CGP concentration used (determined using a monoexponential association equation; Eq. 1). Specific dissociation data were normalized to the fluorescence intensity level measured before initiation of imaging buffer only perfusion. *C* and *D*) Single cell analysis of observed association rates (*C*) and dissociation rate (*D*) constants for 10, 30, and 100 nM BODIPY-TMR-CGP, with each replicate representing the kinetic parameter of 1 single cell. Data shown are means ± sem of 50, 60, and 40 separate cells for 10, 30, and 100 nM BODIPY-TMR-CGP, respectively, and represent the parameter estimates of single cells from 5, 6, and 4 separate experiments for 10, 30, and 100 nM BODIPY-TMR-CGP, respectively. Summary data and statistical analysis are provided in Table 1.

The association of 10, 30, and 100 nM BODIPY-TMR-CGP in CHO-β_1_ cells was monoexponential, and a plateau of BODIPY-TMR-CGP binding was reached following 4.5-min exposure of CHO-β_1_ cells to 30 and 100 nM, but not 10 nM, BODIPY-TMR-CGP (Fig. 3*C*). The concentration dependence of the observed association rates of 10, 30, and 100 nM BODIPY-TMR-CGP at the β_1_-adrenoceptor was clearly seen for the normalized group data (**Fig. 3*A***), as well as the individual cell data (Fig. 3*C*). The derived observed association rates (*k*_onobs_) increased from 0.63 ± 0.08 (*n* = 5) to 1.64 ± 0.08 (*n* = 6) and 3.08 ± 0.11 min^−1^ (*n* = 4) for 10, 30, and 100 nM BODIPY-TMR-CGP, respectively. The dissociation of 10 nM BODIPY-TMR-CGP was also monoexponential yielding a dissociation rate of 0.09 ± 0.01 min^−1^ (*n* = 5). By contrast, 2 dissociation components, a fast (*k*_off(fast)_) and a slow (*k*_off(slow)_) component, were detected in the dissociation of 30 and 100 nM BODIPY-TMR-CGP. The rate of the fast dissociating component was comparable to the dissociation rate determined in CHO-CS cells and was thus defined as a nonspecific component, which was constrained to the average dissociation rate obtained in CHO-CS cells (2.47 min^−1^) in the fitting of the 2-component dissociation rates. The slow dissociation component determined for 30 and 100 nM BODIPY-TMR-CGP in CHO-β_1_ cells was 0.09 ± 0.01 (*n* = 6) and 0.14 ± 0.02 min^−1^ (*n* = 4), respectively. Interestingly, the dissociation rate determined for 100 nM BODIPY-TMR-CGP was significantly faster than the rate determined for 10 and 30 nM BODIPY-TMR-CGP (*P* < 0.05, 1-way ANOVA followed by Tukey’s multiple comparisons test; Table 1). We have previously shown that 100 nM BODIPY-TMR-CGP binds to the secondary low affinity conformation of the β_1_-adrenoceptor, and as such may cause allosteric effects that result in an enhanced dissociation rate of 100 nM BODIPY-TMR-CGP (*i.e.,* negative cooperativity) from the primary high-affinity β_1_-adrenoceptor conformation. This was not evident for 10 and 30 nM BODIPY-TMR-CGP, which is likely caused by lower occupancy levels of these BODIPY-TMR-CGP concentrations at the secondary β_1_-adrenoceptor conformation compared with 100 nM BODIPY-TMR-CGP (24). The concentration independence of the dissociation rates of 10 and 30 nM BODIPY-TMR-CGP and the concentration dependence of the observed association rates of 10, 30, and 100 nM BODIPY-TMR-CGP at the β_1_-adrenoceptor were clearly seen for normalized grouped data (Fig. 3*A, B*), as well as single cell data (Fig. 3*C, D*). The observed association (*k*_onobs_) and β_1_-adrenoceptor–specific dissociation rate (*k*_off(slow)_) of each BODIPY-TMR-CGP concentration were used to determine the association rate constants (*k*_on_). The *k_on_* and *k*_off(slow)_ were then used to calculate the equilibrium dissociation constant (*K_D_*) for each BODIPY-TMR-CGP concentration and are summarized in Table 1.

### Cooperative interactions between the high- and low-affinity conformation of the human β_1_-adrenoceptor

The dissociation rate of a ligand should not be altered in the presence of a second ligand if the two compete for the same binding site. However, if the second ligand binds to a separate second binding site, a resulting conformational change could lead to cooperative (allosteric) effects and thus affect the dissociation rate of the first ligand (23, 28–30). To assess whether the faster dissociation rate observed for 100 nM BODIPY-TMR-CGP was in fact caused by cooperative interactions between the high- and low-affinity conformation of the β_1_-adrenoceptor, we investigated the dissociation rates of 3 nM BODIPY-TMR-CGP in the absence and presence of increasing concentrations of CGP 12177 and propranolol. We previously showed that 3 nM BODIPY-TMR-CGP predominantly labels the high-affinity conformation over the secondary low-affinity conformation of the β_1_-adrenoceptor [∼86% and 3% occupancy, respectively, based on the affinity of BODIPY-TMR-CGP for the high- and low-affinity β_1_-adrenoceptor conformations determined in functional assays (24)]. This limits competition of labeled and unlabeled ligands at the secondary β_1_-adrenoceptor conformation and therefore ensures that any observed effects on the BODIPY-TMR-CGP dissociation rate are caused by the unlabeled ligand used.

**Figure 4.**
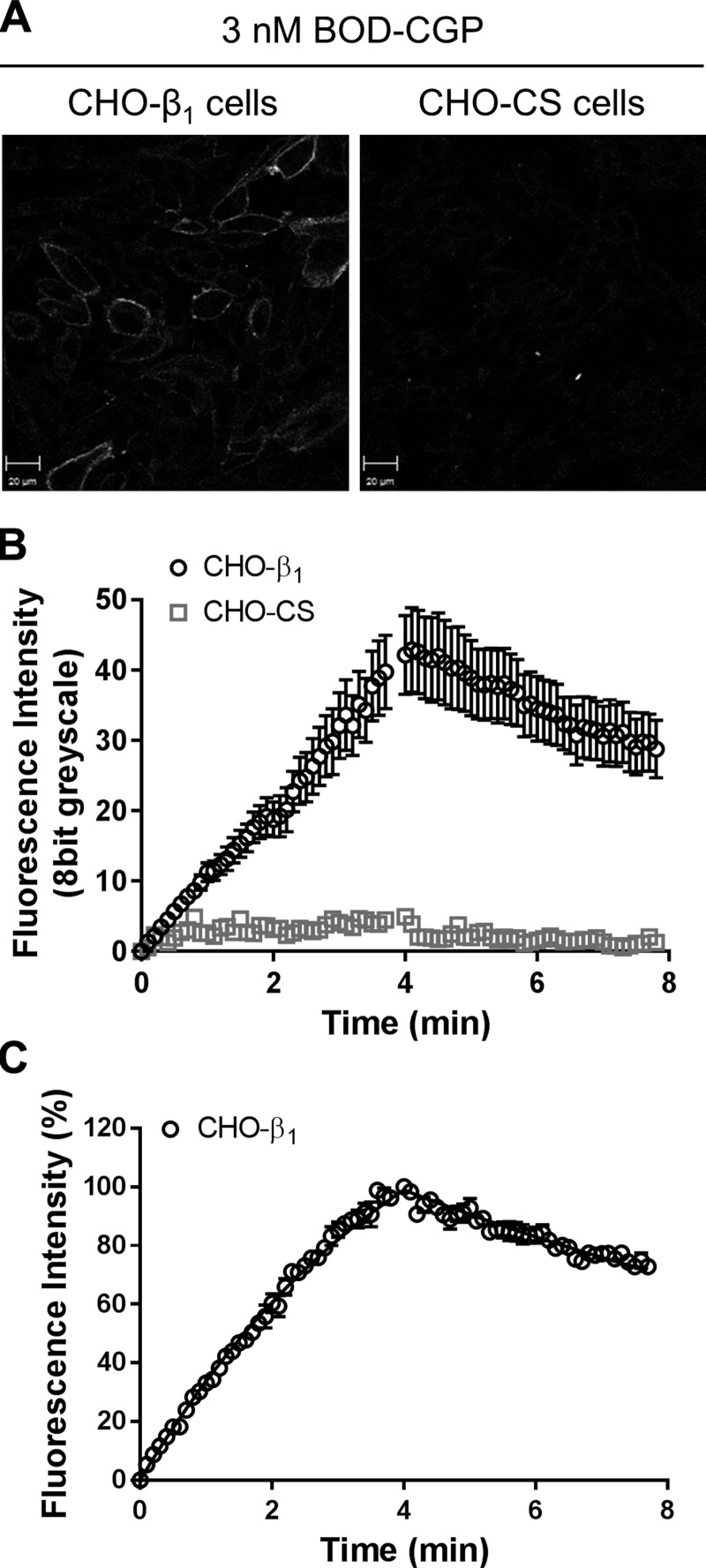
Association and dissociation kinetics of 3 nM BODIPY-TMR-CGP in CHO-β_1_ and CHO-CS cells. *A*) Confocal images show BODIPY-TMR-CGP (BOD-CGP) binding to CHO-β_1_ and CHO-CS cells after 4 min BODIPY-TMR-CGP association and are representative of 9 and 4 experiments for CHO-β_1_ and CHO-CS cells, respectively. Scale bars, 20 μm. *B*) Association and dissociation kinetics of 3 nM BODIPY-TMR measured in CHO-β_1_ and CHO-CS cells. Data shown are means ± sem of fluorescence intensities of 10 cells measured every 2 s (shown every 6 s for better visualization) in a single experiment that is representative of 9 and 4 experiments for CHO-β_1_ and CHO-CS cells, respectively. *C*) Normalized association and dissociation of 3 nM BODIPY-TMR-CGP at CHO-β_1_ cells. Data were normalized to the BODIPY-TMR-CGP binding level following 4 min association to allow data to be grouped and are means ± sem of 9 separate experiments. Each experimental replicate reflects the average fluorescent intensity of the plasma membrane of 5–10 single cells.

First, we assessed the association and dissociation kinetics of 3 nM BODIPY-TMR-CGP at CHO-β_1_ and CHO-CS cells (**Fig. 4**). The fluorescence intensities measured in CHO-CS cells were too low to accurately determine observed association and dissociation rates. In line with this, the nonspecific binding component in the 3 nM BODIPY-TMR-CGP dissociation trace obtained in CHO-β_1_ cells was also too low to be detected and therefore was analyzed as β_1_-adrenoceptor-specific dissociation using a 1-phase dissociation equation. This gave a dissociation rate of 0.09 ± 0.01 min^−1^ (*n* = 9) in the absence of unlabeled ligands, which was similar to the dissociation rate obtained for 10 and 30 nM BODIPY-TMR-CGP (*P* > 0.05, 1-way ANOVA followed by Tukey’s multiple comparisons test). To selectively label the high-affinity conformation of the β_1_-adrenoceptor, we chose a concentration of 3 nM BODIPY-TMR-CGP (24) for subsequent dissociation experiments and limited the association of fluorescent ligand to 4 min. As a consequence, the association of 3 nM BODIPY-TMR-CGP did not reach a plateau within this time period, and the association rate could not be accurately determined from these data alone. However, when globally analyzed in conjunction with the dissociation trace, an association rate constant (*k*_on_) of 5.27 ± 0.53 × 10^7^ M^−1^⋅min^−1^ (*n* = 9), a dissociation rate (*k*_off_) of 0.08 ± 0.01 min^−1^ (*n* = 9), and a p*K_D_* of 8.83 ± 0.06 (*n* = 9) were obtained (Fig. 4*C*).

**Figure 5.**
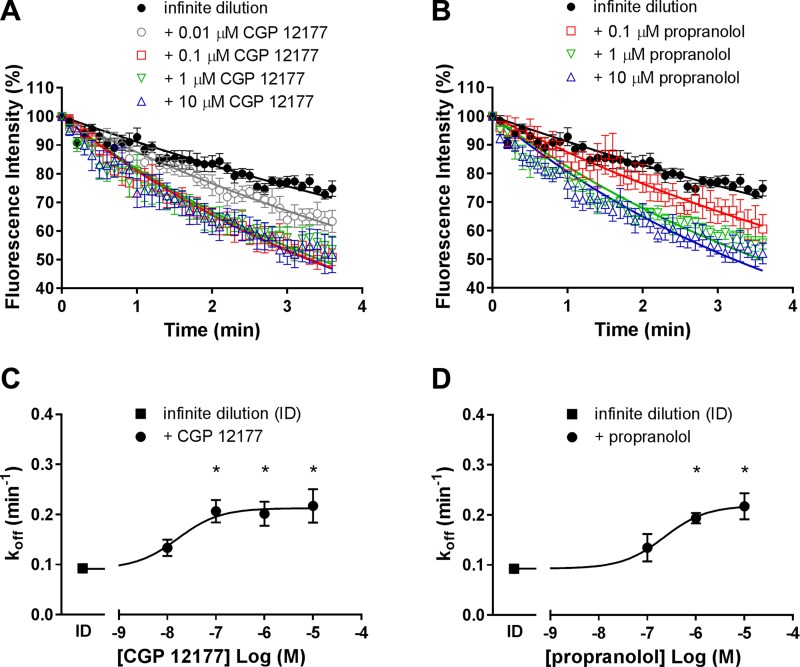
Enhanced dissociation of 3 nM BODIPY-TMR-CGP in CHO-β_1_ cells in the presence of CGP 12177 and propranolol. 3 nM BODIPY-TMR-CGP dissociation was monitored under ID conditions every 2 s (shown every 6 s for better visualization) in the absence (*n* = 9) of unlabeled ligands and in the presence of (*A*) 10 nM (*n* = 6), 100 nM (*n* = 5), 1 μM (*n* = 7), and 10 μM (*n* = 5) CGP 12177 or (*B*) 100 nM (*n* = 3), 1 µM (*n* = 6), and 10 µM (*n* = 5) propranolol. Data were normalized to the fluorescence intensity level measured before initiation of imaging buffer only perfusion. The lower panels show the change in dissociation rate constant (*k*_off_) of 3 nM BODIPY-TMR-CGP in the absence and presence of increasing CGP 12177 (*C*) or propranolol (*D*). The midpoint of the concentration-effect curves provided the apparent CGP 12177 and propranolol affinities for the secondary binding site through which the cooperative effects were mediated. All data shown are means ± sem of indicated *n* separate experiments. Each experimental replicate reflects the average fluorescent intensity of the plasma membrane of 5–10 cells. *Statistical significance (*P* < 0.05) from control conditions (ID) as determined by 1-way ANOVA followed by Dunnett’s multiple comparisons test.

Next, the influence of unlabeled ligands on the BODIPY-TMR-CGP dissociation rate was examined, and the dissociation rate of 3 nM BODIPY-TMR-CGP was significantly enhanced in the presence of 100 nM (0.21 ± 0.02 min^−1^, *n* = 5), 1 µM (0.20 ± 0.02 min^−1^, *n* = 7), and 10 µM (0.22 ± 0.03 min^−1^, *n* = 5) CGP 12177 (*P* < 0.05, 1-way ANOVA followed by Dunnett’s multiple comparisons test; **Fig. 5*A*** and **Table 2**). A similar increase in BODIPY-TMR-CGP dissociation rate was observed in the presence of 1 μM (0.19 ± 0.01 min^−1^, *n* = 6) and 10 µM (0.22 ± 0.03 min^−1^, *n* = 5) propranolol (Fig. 5*B*). The effect of the enhanced dissociation rate was concentration dependent and saturable, which is characteristic of allosteric interactions (28). A concentration-response curve was fitted through the grouped BODIPY-TMR-CGP dissociation rates (listed in Table 2) plotted against the concentrations of unlabeled CGP 12177 (Fig. 5*C*) and propranolol (Fig. 5*D*) used, with the midpoint of the curve providing affinity estimates (*pK_D_*) of the unlabeled ligands for a secondary conformation on the β_1_-adrenoceptor with the fluorescent ligand already bound to the primary orthosteric conformation, which were determined to be 7.79 and 6.65 for CGP 12177 and propranolol, respectively. These values are consistent with those determined from inhibition of functional CGP 12177 responses via the secondary conformation of the β_1_-adrenoceptor (11, 24).

**TABLE 2.** Dissociation rate constants of 3 nM BODIPY-TMR-CGP in the absence and presence of CGP 12177 and propranolol

Condition	CGP 12177		Propranolol	
	*k*_off_ (min^−1^)	*n*	*k*_off_ (min^−1^)	*n*
CHO-β_1_				
Infinite dilution	0.09 ± 0.01	9	0.09 ± 0.01	9
+10 nM	0.13 ± 0.02	6	ND	
+100 nM	0.21 ± 0.02*	5	0.13 ± 0.03	3
+1 µM	0.20 ± 0.02*	7	0.19 ± 0.01*	6
+10 µM	0.22 ± 0.03*	5	0.22 ± 0.03*	5
CHO-β_1_TM4				
Infinite dilution	0.07 ± 0.02	8	0.07 ± 0.02	8
+1 µM	0.09 ± 0.01^#^	6	0.08 ± 0.02^#^	10
CHO-β_1_YFP_N/_β_1_YFP_C_				
Infinite dilution	0.02 ± 0.01^#^	5	0.02 ± 0.01^#^	5
+1 µM	0.19 ± 0.01^#^^,^*^@^*	6	0.19 ± 0.01^#^^,^*^@^*	6
CHO-β_1_YFP_N/_β_1D138A_YFP_C_				
Infinite dilution	0.05 ± 0.01	5	0.05 ± 0.01	5
+1 µM	0.17 ± 0.01^#^^,^*^@^*	4	0.14 ± 0.01^#^^,^*^@^*	5

Data are means ± sem of *n* separate experiments. ND, not determined. *Statistical significance (*P* < 0.05) in CHO-β_1_ cells from control conditions (infinite dilution) for each unlabeled ligand used (1-way ANOVA followed by Dunnett’s multiple comparisons test). ^#^Statistical significance (*P* < 0.05) from the equivalent value in CHO-β_1_ cells and ^@^*P* < 0.05 from control conditions (infinite dilution) within each cell line (2-way ANOVA followed by Tukey’s multiple comparisons test).

### Influence of TM4 on cooperative interactions involving the β_1_-adrenoceptor

**Figure 6.**
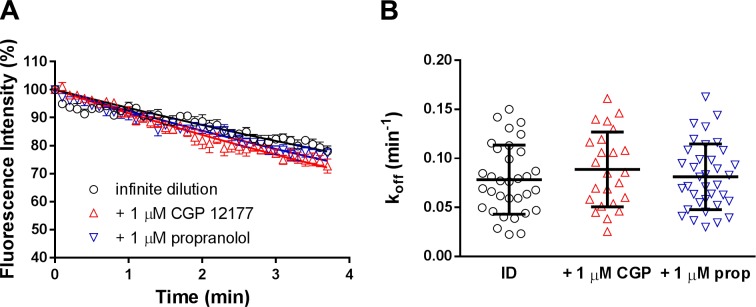
Role of TM4 of the β_1_-adrenoceptor in the observation of cooperative interactions between BODIPY-TMR-CGP and CGP 12177 and propranolol. Dissociation of 3 nM BODIPY-TMR-CGP was measured under infinite dilution conditions every 2 s (shown every 6 s for better visualization) in CHO-β_1_TM4 cells stably expressing β_1_-adrenoceptors in which its native TM4 region was mutated to be the TM4 region of the β_2_-adrenoceptor. *A*) Normalized 3 nM BODIPY-TMR-CGP dissociation in the absence (*n* = 8) and presence of 1 μM CGP 12177 (*n* = 6) or 1 μM propranolol (*n* = 10), with data shown as means ± sem of the indicated *n* separate experiments. Each experimental replicate reflects the average fluorescent intensity of the plasma membrane of 3–6 single cells within a single experiment. Data were normalized to the fluorescence intensity level measured before initiation of imaging buffer only perfusion. *B*) Single cell dissociation rate constants (*k*_off_) for 3 nM BODIPY-TMR-CGP under infinite dilution conditions in the absence (ID; *n* = 33) and presence of 1 μM CGP 12177 (CGP; *n* = 24) and 1 μM propranolol (prop; *n* = 36), with each *n* representing the number of individual cells measured. Solid lines show the means ± sem of indicated number of single cells obtained from 8 (ID), 6 (CGP 12177), and 10 (propranolol) separate experiments.

To further investigate the cooperative effects observed above on the BODIPY-TMR-CGP dissociation rate from the catecholamine conformation by the action of propranolol and CGP 12177 acting at the secondary conformation, we examined the effect of CGP 12177 on the BODIPY-TMR-CGP dissociation kinetics in CHO-β_1_TM4 cells. These cells express β_1_-adrenoceptors that have been mutated such that the residues in TM4 are those of the β_2_-adroceptor (11). Importantly, this mutant β_1_-adrenceptor does not exhibit the secondary CGP 12177 conformation (11). In the absence of unlabeled ligands, the dissociation rate of 3 nM BODIPY-TMR-CGP in CHO-β_1_ TM4 cells was 0.066 ± 0.005 min^−1^ (*n* = 8) in these cells. Interestingly, the dissociation rates of 3 nM BODIPY-TMR-CGP in the presence of 1 µM CGP 12177 and 1 µM propranolol were 0.089 ± 0.010 (*n* = 6) and 0.080 ± 0.007 min^−1^ (*n* = 10), respectively, which are comparable to the dissociation rate in the absence of ligands (*P* > 0.05, 1-way ANOVA followed by Dunnett’s multiple comparisons test; **Fig. 6**).

### Effects of enhancing and disrupting β_1_AR homodimer interactions on cooperative interactions

The cooperative effects observed above at the wild-type β_1_-adrenoceptor clearly highlight the presence of 2 distinct binding conformations to which β-adrenoceptor ligands can bind. Baker *et al.* (7) conducted mutagenesis studies in which selected mutations (*e.g.,* D138A) in the orthosteric ligand binding domain disrupted both the high- and low-affinity binding conformation (7). Two key mutations in the β_1_-adrenoceptor TM4 region completely abolished the secondary conformation (L195Q and W199Y) (11). Interestingly, these are thought to lie within the TM4-TM5 heterodimer interface of the β_1_-adrenoceptor (19), suggesting a potential role of β_1_-adrenoceptor homodimerization in the secondary conformation CGP 12177 pharmacology.

Furthermore, this study showed that the cooperative interactions at the wild-type β_1_-adrenoceptor are completely prevented in CHO-β_1_TM4 cells (Fig. 6). Homodimerization of β_1_-adrenoceptors has been reported to be transient (20, 21). Thus, to detect β_1_-adrenoceptor homodimers and allow their pharmacological investigation, we used BiFC (31, 32) to irreversibly trap and stabilize β_1_-adrenoceptor homodimers that formed at any given time. We hypothesized that constraining dimers using BiFC would increase the percentage of β_1_-adrenoceptors dimers and as such enhance any dimer-mediated allosteric effects. It has been shown previously that a D138A mutation in TM3 disrupted ligand binding to both the catecholamine and the secondary conformation of the β-adrenoceptor (7). Consequently, any enhanced allosteric effects should be prevented by constraining dimers containing 1 nonligand-binding protomer (β_1D138A_) (7).

**Figure 7.**
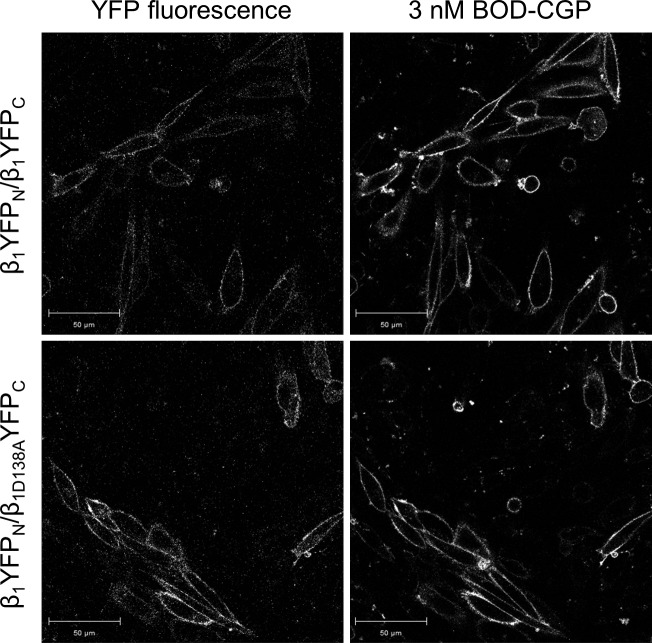
Cell surface expression of BiFC-constrained β_1_-adrenoceptor homodimers. Confocal images show binding of 3 nM BODIPY-TMR-CGP following 4-min association to CHO-K1 cells transiently expressing BiFC-constrained wild-type β_1_-adrenoceptor homodimers (β_1_YFP_N_/β_1_YFP_C_; upper panel) and BiFC-constrained β_1_-adrenoceptor homodimers containing 1 wild-type and 1 nonligand-binding protomer (β_1_YFP_N_/β_1D138A_YFP_C_; lower panel). The fluorescence of the complimented YFP was measured simultaneously to confirm the cell surface expression of β_1_-adrenoceptor homodimers. Images are representative of 5 separate experiments. Scale bars, 50 μm. The binding of BODIPY-TMR-CGP in the lower right confirms that the β_1_YFP_N_/β_1D138A_YFP_C_ dimer pairs contain a viable single orthosteric binding site.

**Figure 8.**
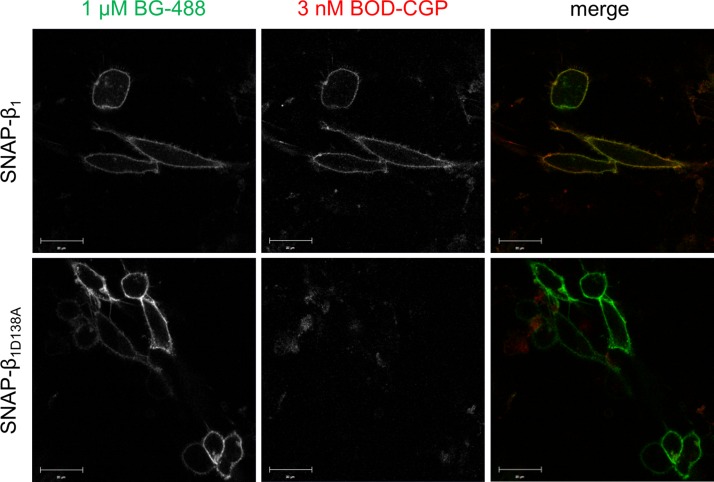
Cell surface expression of the nonligand-binding β_1D138A_-adrenoceptor. The cell surface expression of SNAP-tagged wild-type (SNAP-β_1_; upper panel) and nonligand-binding (SNAP-β_1D138A_; lower panel) β_1_-adrenoceptor was visualized following labeling of the SNAP-tag with 1 μM BG-488 (30 min, 37°C). The ligand-binding properties of the SNAP-tagged wild-type and nonligand-binding receptors were examined by exposing the cells to 3 nM BODIPY-TMR-CGP (10 min, 37°C). The merged images highlight colocalization of cell surface SNAP-tagged β_1_-adrenoceptors (green) and BODIPY-TMR-CGP (BOD-CGP) binding (red) to these receptors in yellow pixels. The images are representative of 3 different fields of view imaged on 3 separate experimental days. Scale bars, 20 μm.

BiFC uses 2 nonfluorescent fragments of a fluorescent protein, which reconstitute the functional (*i.e.,* fluorescent) full-length fluorescent protein when in close proximity to one another (32). The N-terminal fragment and the C-terminal fragment of the YFP (YFP_N_ and YFP_C_, respectively) were fused to the C-terminal end of the wild-type or D138A β_1_-adrenoceptor to generate the β_1_YFP_N_, β_1_YFP_C_, and β_1D138A_YFP_C_ receptor constructs. The β_1_YFP_N_/β_1_YFP_C_ and β_1_YFP_N_/β_1D138A_YFP_C_ constructs were transiently cotransfected into CHO-K1 cells, and clear membrane fluorescence of reconstituted YFP and BODIPY-TMR-CGP binding could be seen (**Fig. 7**), confirming cell surface expression of wild-type/wild-type and wild-type/D138A β_1_-adrenoceptor homodimers that each contain at least 1 BODIPY-TMR-CGP binding conformation. To confirm that the D138A mutation abolished ligand binding to the β_1_-adrenoceptor, we examined the binding of 3 nM BODIPY-TMR-CGP to a SNAP-tagged D138A β_1_-adrenoceptor. Indeed, no binding of 3 nM BODIPY-TMR-CGP could be seen in CHO-K1 cells transiently transfected with the SNAP-β_1D138A_ construct, but clear membrane fluorescence was observed following labeling of the SNAP-tag with 1 μM BG-488, confirming the expression of the non–ligand-binding receptor at the cell surface (**Fig. 8**, lower left panel). A SNAP-tagged wild-type β_1_-adrenoceptor was transiently transfected as a positive control, and clear fluorescence of the BG-488 labeled SNAP-tag and 3 nM BODIPY-TMR-CGP binding to the wild-type receptor can be seen (Fig. 8, upper panel). This indicates that the lack of BODIPY-TMR-CGP fluorescence seen for the SNAP-β_1D138A_–transfected cells is caused by the D138A mutation introduced into the β_1_-adrenoceptor.

**Figure 9.**
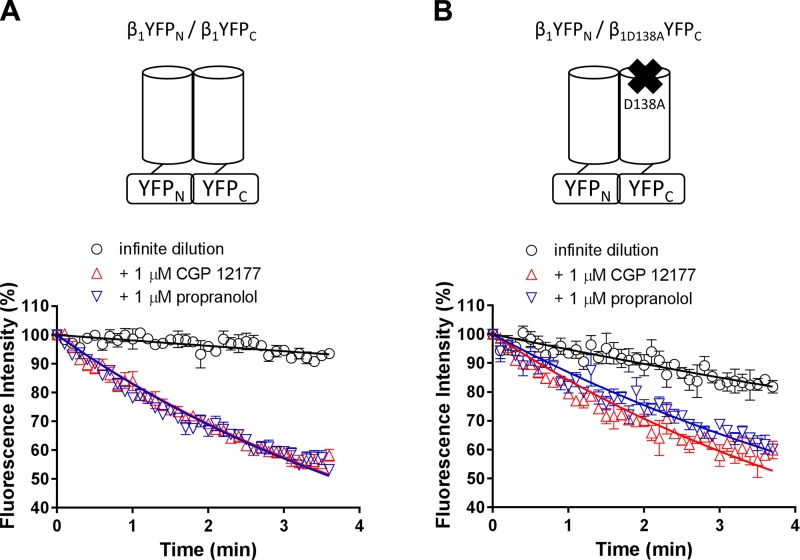
Cooperative interactions between BODIPY-TMR-CGP and CGP 12177 and propranolol in bimolecular fluorescence complementation-constrained β_1_-adrenoceptor dimers. Dissociation of 3 nM BODIPY-TMR-CGP was measured under ID conditions every 2 s (shown every 6 s for better visualization). *A*) Normalized dissociation of 3 nM BODIPY-TMR-CGP in CHO-K1 cells transiently expressing wild-type (β_1_YFP_N_/β_1_YFP_C_) BiFC-constrained β_1_-adrenoceptor homodimers under ID conditions in the absence (*n* = 5) and presence of 1 μM CGP 12177 (*n* = 6) and 1 μM propranolol (*n* = 6). *B*) Normalized dissociation of 3 nM BODIPY-TMR-CGP in CHO-K1 cells transiently expressing 1 wild-type and 1 nonligand-binding (β_1_YFP_N_/β_1D138A_YFP_C_) protomer in BiFC-constrained β_1_-adrenoceptor homodimers under ID conditions in the absence (*n* = 5) and presence of 1 μM CGP 12177 (*n* = 6) and 1 μM propranolol (*n* = 5). Data were normalized to the fluorescence intensity level measured before initiation of imaging buffer only perfusion and show means ± sem of the indicated *n* separate experiments. Each experimental replicate reflects the average fluorescent intensity of the plasma membrane of 10 cells.

We then examined the dissociation rate of 3 nM BODIPY-TMR-CGP at irreversibly constrained stable wild-type/wild-type β_1_-adrenoceptor homodimers under ID conditions, which was determined to be 0.02 ± 0.01 min^−1^ (*n* = 5; **Fig. 9*A***). This was significantly slower than the dissociation rate measured in CHO-β_1_ cells (*P* < 0.05, 2-way ANOVA analysis followed by Tukey’s multiple comparisons test). The dissociation of 3 nM BODIPY-TMR-CGP binding was enhanced in the presence of 1 µM CGP 12177 and 1 µM propranolol with dissociation rates of 0.186 ± 0.008 (*n* = 6) and 0.189 ± 0.007 min^−1^ (*n* = 6), respectively (Fig. 9*A*). This was significantly faster than the dissociation rate determined in the absence of unlabeled ligands (*P* < 0.05, 2-way ANOVA followed by Tukey’s multiple comparisons test; Fig. 9*A*). The change in the 3 nM BODIPY-TMR-CGP dissociation rate in the absence and presence of unlabeled ligands was ∼2-fold in CHO-β_1_ cells but was ∼10-fold in CHO-K1 cells expressing β_1_YFP_N_/β_1_YFP_C_ homodimers.

The transient expression of BiFC constructs yielded a mixed population of cells with different expression levels (Fig. 7 and Supplemental Fig. S1). To investigate whether higher expression levels of β_1_-adrenoceptor homodimers affected the BODIPY-TMR-CGP dissociation rate measurements in this study, we compared the measurements taken in cells with both high and low expression levels (Supplemental Fig. S1). These data confirmed that the dissociation kinetics were very similar at both expression levels (Supplemental Fig. S1).

The dissociation rate of 3 nM BODIPY-TMR-CGP in CHO-K1 cells transiently transfected with 1 wild-type and 1 nonligand-binding β_1_-adrenoceptor construct (β_1_YFP_N_/β_1D138A_YFP_C_) under ID conditions was determined to be 0.054 ± 0.011 min^−1^ (*n* = 5). This dissociation rate was increased in the presence of 1 µM CGP 12177 (*k*_off_, 0.169 ± 0.010 min^−1^; *n* = 6) and 1 µM propranolol (*k*_off_, 0.144 ± 0.009 min^−1^; *n* = 5; *P* < 0.05, 2-way ANOVA followed by Tukey’s multiple comparisons test; Fig. 9*B*). Interestingly, the difference in the 3 nM BODIPY-TMR-CGP dissociation rate in the presence of unlabeled ligands compared with in the absence of unlabeled ligands was ∼3-fold in cells expressing constrained wild-type/nonligand-binding β_1_-adrenoceptor homodimers.

## DISCUSSION

In this study, we used a confocal microscopy approach in conjunction with a perfusion system to investigate the dissociation kinetics of BODIPY-TMR-CGP from the human β_1_-adrenoceptor under ID conditions in single living cells. Using this technique, we revealed negative cooperative interactions between the high- and low-affinity β_1_-adrenoceptor conformations, which are facilitated by β_1_-adrenoceptor homodimerization.

In CHO-β_1_ cells, the observed association rates for 10, 30, and 100 nM BODIPY-TMR-CGP increased in a concentration-dependent manner. The dissociation rate was monophasic for 10 nM fluorescent ligand, but biphasic for higher concentrations (30 and 100 nM) of BODIPY-TMR-CGP. The fast dissociation component was clearly identified as a nonspecific component as it was comparable to the dissociation rate observed in CHO-CS cells lacking the β_1_-adrenoceptor. The β_1_-adrenoceptor–specific dissociation rates were comparable for 10 and 30 nM BODIPY-TMR-CGP, but not for 100 nM BODIPY-TMR-CGP, where a significantly faster dissociation rate was observed. According to classic receptor theory, dissociation rates of a ligand should be independent of the ligand concentration used. However, this analysis assumes that the ligand only binds to 1 receptor binding conformation (25). We recently described BODIPY-TMR-CGP binding to both the high- and low-affinity conformations of the human β_1_-adrenoceptor with affinity values of 0.6 and 87 nM, respectively (24). Thus, the observation of a faster dissociation rate for 100 nM BODIPY-TMR-CGP may be caused by BODIPY-TMR-CGP binding to both the high- and low-affinity β_1_-adrenoceptor conformations and may be a consequence of negative cooperativity occurring between these 2 ligand-bound conformations.

To further explore the potential for negative cooperativity between different conformations within the β_1_-adrenoceptor, we examined the dissociation rate of 3 nM BODIPY-TMR-CGP in the absence and presence of increasing concentrations of unlabeled ligands. The affinity value derived from the association and dissociation parameters obtained for 3 nM BODIPY-TMR-CGP (∼2.6 nM) compared well to the affinity value of BODIPY-TMR-CGP for the orthosteric binding conformation determined in functional studies (0.6 nM) (24). This strongly suggests that 3 nM BODIPY-TMR-CGP predominantly binds the high-affinity β_1_-adrenoceptor conformation. This therefore allows potential cooperative effects exerted by unlabeled ligands binding to a secondary conformation to be measured. The dissociation rate of a labeled ligand should remain unchanged in the presence of an unlabeled ligand that binds to the same receptor site (28). Therefore, any changes of the dissociation rate of 3 nM BODIPY-TMR-CGP from the high-affinity β_1_-adrenoceptor conformation would be caused by the unlabeled β-adrenoceptor ligands CGP 12177 and propranolol binding to a topographically distinct (allosteric) binding conformation that exerts a cooperative effect on the high-affinity (orthosteric) conformation. Indeed, the BODIPY-TMR-CGP dissociation rate was enhanced by 100 nM CGP 12177 and propranolol (and concentrations above), clearly highlighting the presence of a secondary topographically distinct β_1_-adrenoceptor binding conformation through which CGP 12177 and propranolol exert negative cooperative effects on the primary high-affinity β_1_-adrenoceptor conformation. The apparent dissociation constants (*K_D_*) of CGP 12177 and propranolol for this secondary (allosteric) conformation could be determined from the concentration dependence of the cooperative effect of each ligand on the BODIPY-TMR-CGP dissociation rates. These were similar to those values determined for these ligands for the low-affinity β_1_-adrenoceptor conformation in previous functional studies (24). This allosteric conformation could therefore be responsible for the secondary low-affinity CGP 12177 β_1_-adrenoceptor conformation.

A previous mutagenesis study by Baker *et al.* (7) reported that the 2 β_1_-adrenoceptor conformations must overlap to some degree, as the introduction of certain single point mutations (*e.g.,* D138A) in the β_1_-adrenoceptor affected both conformations. However, Baker *et al.* (11) recently identified key residues in TM4 that only affect the pharmacology of the secondary low-affinity β_1_-adrenoceptor conformation, leaving the catecholamine conformation unaffected. The same mutations in TM4 also removed the cooperative effect observed in this study (Fig. 6).

Furthermore, this clearly highlights that the BODIPY-TMR-CGP dissociation kinetics measured in this study were not influenced by rebinding of the fluorescent ligand to unoccupied β_1_-adrenoceptors on neighboring cells. Rebinding can potentially result in a slowed dissociation rate of the labeled ligand, which is then increased in the presence of a high concentration of a competing ligand that prevents rebinding of the labeled ligand (33, 34). In this study, the rapid fluid exchange of the perfusion system was used to prevent the reassociation of the fluorescent ligand (27). Most convincingly, however, high concentrations of unlabeled CGP 12177 and propranolol that in CHO-β_1_TM_4_ cells are strictly competitive to BODIPY-TMR-CGP binding did not cause an increased dissociation rate of BODIPY-TMR-CGP.

**Figure 10.**
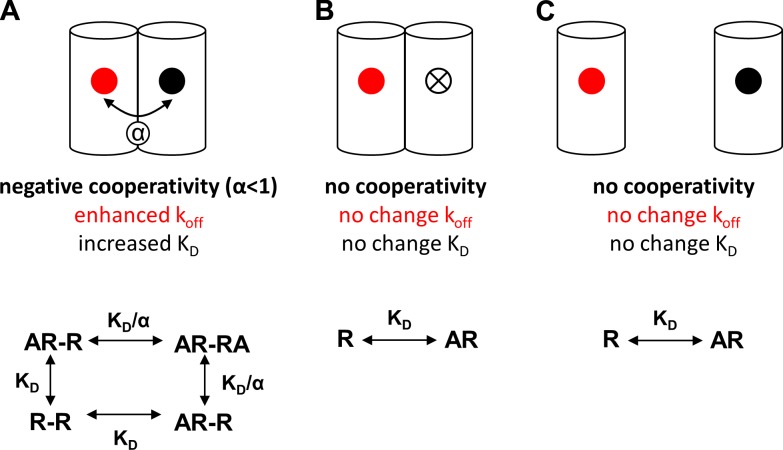
Schematic diagram describing negative cooperative interactions at β_1_-adrenoceptor homodimers. *A*) A β_1_-adrenoceptor homodimer possesses 2 endogenous (*i.e.,* structurally identical) ligand-binding sites for which β_1_-adrenoceptor ligands have the same affinity. However, following binding of a β_1_-adrenoceptor ligand to 1 β_1_-adrenoceptor site (primary, orthosteric binding site; red filled circles) with high affinity, negative cooperativity between the 2 β_1_-adrenoceptor binding sites in a β_1_-adrencoeptor homodimer results in a markedly reduced affinity (increased *K*_D_) of a secondary β-adrenoceptor ligand for the secondary β_1_-adrenoceptor site (secondary, allosteric binding site; black filled circles), which is often described as the low-affinity CGP 12177 binding site. This negative cooperativity is reciprocal between the 2 β_1_-adrenoceptor binding sites, and ligand binding to the secondary site causes an enhanced dissociation rate of the ligand already bound to the primary site. The cooperativity factor α provides a quantitative estimate of the degree and direction of cooperativity between 2 binding sites and is defined as the ratio of a ligand’s affinity for the free receptor over the affinity of the same ligand for the already ligand-occupied receptor (23, 28). Negative cooperativity is indicated by a cooperativity factor smaller than unity. The low-affinity β_1_-adrenoceptor site therefore represents the binding affinity of ligand A for a receptor dimer where 1 protomer is already occupied, and the dissociation constant is given by *K*_D_/α (see equation). Effective removal of the orthosteric site from one of the protomers through (*B*) a point mutation that abolishes ligand binding or (*C*) disruption of the TM4/TM5 β_1_-adrenoceptor dimerization interface removes any cooperative effects between 2 β_1_-adrenoceptor sites across the homodimer interface. In these 2 situations, the binding equation reverts to a simple mass action equilibrium between ligand A and receptor R.

The TM4 has been highlighted to play a role in dimerization of various class A GPCRs (35), including the β_1_-adrenoceptor (19, 36), indicating a potential role of β_1_-adrenoceptor homodimerization in the secondary conformation. Homodimers of β_1_-adrenoceptors have been reported to be transient in nature (20). In an attempt to generate more stable β_1_-adrenoceptor dimers, we used a BiFC approach to lock β_1_-adrenoceptor homodimers into constrained stable dimers of defined composition. Although BiFC does not affect the rate of homodimerization (26), the prevention of dimer dissociation as a consequence of the irreversible nature of BiFC will increase the percentage of β_1_-adrenoceptors that exist as homodimers. The successful trapping of BiFC-constrained β_1_-adrenoceptor dimers was demonstrated in CHO cells cotransfected with YFP_N_ and YFP_C_-tagged β_1_-adrenoceptor constructs by the clear membrane labeling observed with the reconstituted YFP. In cells expressing these wild-type (β_1_YFP_N_/β_1_YFP_C_) BiFC-constrained β_1_-adrenoceptor homodimers, the BODIPY-TMR-CGP dissociation rate was enhanced ∼10-fold by 1 µM CGP 12177 and 1 µM propranolol. In contrast, only a ∼3-fold difference in dissociation rate was observed in CHO-β_1_ cells (*i.e.,* transient unconstrained dimers), suggesting that the cooperative effects of unlabeled ligands on the dissociation rate of BODIPY-TMR-CGP may be mediated across a β_1_-adrenoceptor homodimer interface (**Fig. 10*A***). It was also notable that the dissociation of 3 nM BODIPY-TMR-CGP from native transient β_1_-adrenoceptor dimers (*k*_off_, 0.09 min^−1^) was faster than that from wild-type BiFC-constrained β_1_-adrenoceptor homodimers (*k*_off_, 0.02 min^−1^). This suggests that the formation of stable homodimers itself leads to significant basal allosteric influences on ligand binding kinetics.

To further test whether the secondary (allosteric) β_1_-adrenoceptor conformation is facilitated by a second β_1_-adrenoceptor protomer in a homodimer complex, BODIPY-TMR-CGP dissociation kinetics were determined in cells expressing BiFC-constrained β_1_-adrenoceptor homodimers of 1 wild-type β_1_-adrenoceptor (β_1_YFP_N_) and 1 protomer containing a point mutation that abolished binding of β-adrenoceptor ligands at both the primary catecholamine and secondary conformations (β_1D138A_YFP_C_) (7). The removal of one of the orthosteric binding conformations in a homodimeric β-adrenoceptor pair should remove the potential for negatively cooperative affects across the dimer interface (Fig. 10*B*). Indeed, in cells expressing β_1_YFP_N_/β_1D138A_YFP_C_ BiFC-constrained homodimers, the effects of CGP 12177 and propranolol on the BODIPY-TMR-CGP dissociation kinetics were reduced and reflected more closely the pharmacology observed in CHO-β_1_ cells (*i.e.,* transient wild-type unconstrained dimers). This residual cooperativity is most likely caused by the cooperative effects that can still occur across transient wild-type β_1_YFP_N_/β_1_YFP_N_ dimers that will be present in the cell population. Two dimerization interfaces have been reported for the β_1_-adrenoceptor involving transmembrane regions 1 and 2 and helix 8 (TM1/TM2/H8) in the first interface and transmembrane region 4 and 5 and intracellular loop 2 (TM4/TM5/ICL2) in the second interface, the latter of which has been described to make structural rearrangements during receptor activation (19). Key residues (L195, W199) identified by Baker *et al.* (11) that are responsible for the secondary conformation lie within the dimer interface region of TM4. Using a stable cell line containing the reported β_1_-adrenoceptor TM4 mutations that result in no secondary conformation (11), we observed no effects on the dissociation rate of 3 nM BODIPY-TMR-CGP in the presence of 1 µM CGP 12177 and 1 µM propranolol. The loss of the enhanced dissociation rate in cells expressing β_1_-adrenoceptor TM4 mutations is consistent with a role of a dimerization interface involving TM4 in the negative cooperativity observed at the β_1_-adrenoceptor (Fig. 10*C*).

In summary, these data suggest that the secondary low-affinity conformation of the β_1_-adrenoceptor may be a consequence of negative cooperative interactions between 2 orthosteric binding conformations within a β_1_-adrenoceptor homodimer. This can then lead to a reduced apparent affinity of ligands for the second protomer of an already ligand-occupied (on the first protomer) dimer [see Supplemental Fig. S1 in May *et al.* (23)]. The contribution of these cooperative interactions is provided by the cooperativity factor α, and the ligand affinity for the already ligand-bound receptor is described as a ratio of the ligand affinity for the unbound receptor and the cooperativity factor α (*K_B_*/α). As such, the cooperativity factor at β_1_-adrenoceptor dimers can be determined by taking the ratio of the apparent *K_D_* value determined for binding to the first protomer (orthosteric β_1_-adrenoceptor conformation 1 *K_D_*, *i.e.,* unbound receptor) and that determined for modifying the dissociation rate of BODIPY-TMR-CGP from conformation 1 by an unlabeled ligand binding to the second protomer (allosteric β_1_-adrenoceptor conformation 2 *K_D_*, *i.e.,* ligand-bound receptor). Negative cooperativity that leads to an increase in the apparent dissociation observed is reflected in a cooperativity factor smaller than unity. Indeed, the cooperativity factors (α) for CGP 12177 and propranolol were estimated to be 0.015 and 0.010, respectively, using the binding affinities for the orthosteric β_1_-adrenoceptor conformation determined in Gherbi *et al.* (24). A mechanistic framework for the secondary β_1_-adrenoceptor conformation based on homodimer formation opens up new insights into the role of dimerization in altering the molecular pharmacology of GPCRs.

## Supplementary Material

Supplemental Data

## Abbreviations

BiFC: bimolecular fluorescence complementation
BODIPY-TMR-CGP (or BOD-CGP): bordifluoropyrromethane-tetramethylrhodamine-(±)CGP 12177
CHO: Chinese hamster ovary
ID: infinite dilution
ROI: region of interest
TM: transmembrane
YFP: yellow fluorescent protein
