# Supplementary material for: Negative cooperativity across β1-adrenoceptor homodimers provides insights into the nature of the secondary low-affinity CGP 12177 β1-adrenoceptor binding conformation
Source: FASEB J. 2015 Apr 2;29(7):2859–71. doi: 10.1096/fj.14-265199 (PMC4478806; doi:10.1096/fj.14-265199)
Supplement: Supplemental Data [file supp_29_7_2859__index.html]

Negative cooperativity across β1-adrenoceptor homodimers provides insights into the nature of the secondary low-affinity CGP 12177 β1-adrenoceptor binding conformation — Negative cooperativity across β1-adrenoceptor homodimers provides insights into the nature of the secondary low-affinity CGP 12177 β1-adrenoceptor binding conformation — Negative cooperativity across β1-adrenoceptor homodimers provides insights into the nature of the secondary low-affinity CGP 12177 β1-adrenoceptor binding conformation — Supplemental Data 

# Negative cooperativity across β1-adrenoceptor homodimers provides insights into the nature of the secondary low-affinity CGP 12177 β1-adrenoceptor binding conformation

## Supplemental Data

**Files in this Data Supplement:**

- Supplemental Data
