# Supplementary material for: Negative cooperativity across β1-adrenoceptor homodimers provides insights into the nature of the secondary low-affinity CGP 12177 β1-adrenoceptor binding conformation
Source: FASEB J. 2015 Apr 2;29(7):2859–71. doi: 10.1096/fj.14-265199 (PMC4478806; doi:10.1096/fj.14-265199)
Supplement: Supplemental Data [file supp_fj.14-265199_Supplemental_Figure1.pdf]

Supplementary Figure

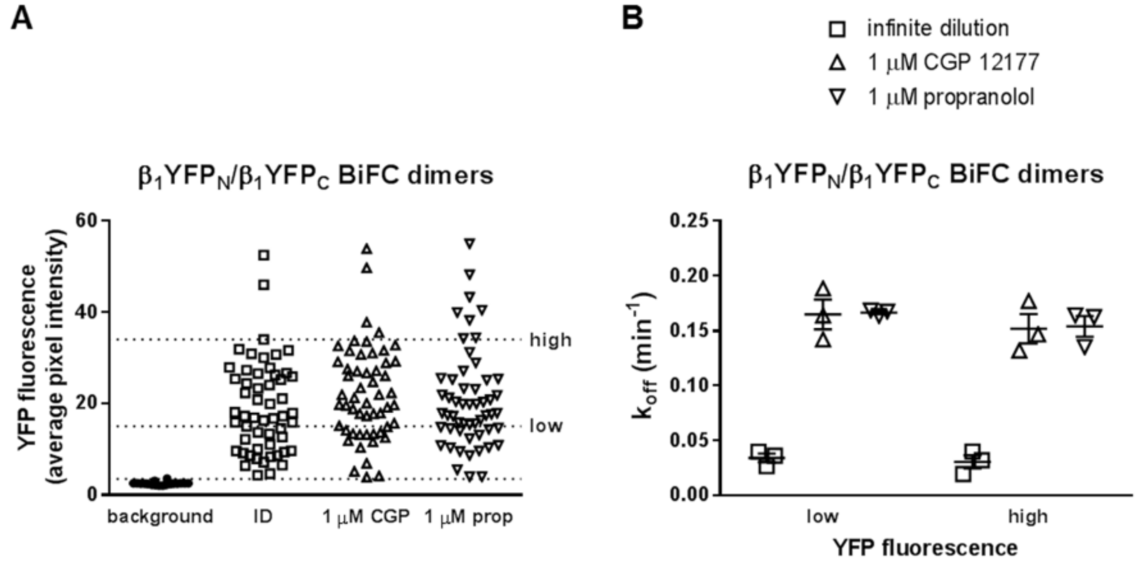

Varying expression levels of receptor homodimers did not affect BODIPY-TMR-CGP dissociation rates. Transient transfection of CHO-K1 cells with BiFC receptor constructs resulted in varying expression levels of BiFC-constrained β_1-adrenoceptor homodimers, which was reflected in different YFP fluorescence intensities. A) Comparable range of YFP fluorescence intensities ranging from just above background fluorescence to near 60 average pixel intensities was observed when detecting $\beta_1YFP_N/\beta_1YFP_C$ homodimers in cells used for measurements of BODIPY-TMR-CGP dissociation rates in the absence (ID, infinite dilution) and presence of 1 µM CGP 12177 (CGP) or 1 µM propranolol (prop). Each replicate represents the YFP fluorescence of a single cell, except for background determinations, which were taken in an area of no cells. B) To examine possible effects of different expression levels of β_1-adrenoceptor homodimers on the measured BODIPY-TMR-CGP dissociation rates in these cells, we compared dissociation rates (k_{off}) of 3 nM BODIPY-TMR-CGP in the absence (infinite dilution) and presence of 1 µM CGP 12177 or 1 µM propranolol in a sample of three cells per condition displaying low and high YFP fluorescence intensities (≤ 15 and ≥ 35 average pixel intensities, respectively; see dotted lines in A) following transfection with $\beta_1YFP_N/\beta_1YFP_C$ receptor constructs. Each replicate represents the dissociation measurements of one single cell, and mean dissociation rates are shown ± s.e.m. with comparable data obtained from cells of low and high YFP fluorescence intensities, suggesting no effect of β_1-adrenoceptor homodimer expression levels on the dissociation rates measured in this study.
